# Retinoblastoma and Its Tumor Microenvironment

**DOI:** 10.3390/curroncol33050264

**Published:** 2026-05-01

**Authors:** Ashwinaa M. Vaithianathan, George Zanazzi

**Affiliations:** 1 Rhodes College, Memphis, TN 38112, USA; 2 Geisel School of Medicine, Dartmouth-Hitchcock Medical Center, Lebanon, NH 03756, USA; george.j.zanazzi@hitchcock.org; 3 Department of Pathology and Laboratory Medicine, Dartmouth-Hitchcock Medical Center, Lebanon, NH 03756, USA; 4 Dartmouth Cancer Center, Dartmouth-Hitchcock Medical Center, Lebanon, NH 03756, USA

**Keywords:** immunotherapy, tumor-associated macrophages, immune checkpoint inhibitors, CAR T-cell, immunosuppression

## Abstract

Retinoblastoma is the most common intraocular malignancy of childhood and is most often driven by loss of the *RB1* tumor suppressor gene. While current treatments achieve high survival rates, they are frequently associated with significant morbidity, highlighting the need for more precise, biology-driven therapeutic methods. Increasing evidence suggests that retinoblastoma progression is not dictated by neoplastic cells alone, but rather by complex interactions within the tumor microenvironment, including stromal and immune components. In this review, we examine the cellular and molecular landscape of retinoblastoma with a particular focus on the immune microenvironment, including the spatial distribution and functional roles of innate and adaptive immune cells, as well as immune checkpoint proteins such as PD-1, PD-L1, and CTLA-4. We discuss how tumor- and treatment-induced immune suppression shapes disease progression and therapeutic response, and how chemotherapy alters immune infiltration and checkpoint expression. Finally, we explore emerging immunotherapeutic and cell-based approaches, emphasizing the potential for combination therapies that integrate immune modulation to improve outcomes and reduce long-term toxicity in retinoblastoma.

## Simple Summary

Retinoblastoma is the most common pediatric ocular neoplasm. Although the genetic underpinnings of this disease have long been studied, some of the contributions of the tumor microenvironment have only recently been elucidated. We review the emerging understanding of retinoblastoma and its tumor microenvironment. Several tumor-associated antigens, such as GD2, are expressed in human retinoblastoma and have shown promise as potential targets for immunotherapy, including CAR T cell therapy. In general, the treatment-naïve tumor microenvironment is immunosuppressive, with M2 polarized tumor-associated macrophages, low CD8+ T cell infiltration and low PD-L1 expression. However, chemotherapy can significantly alter the tumor’s microenvironment, potentially enhancing immune responses. Therefore, emerging combination strategies and cell-based approaches for immunotherapy provide additional options for the treatment of patients with this malignant disease.

## 1. Introduction

Retinoblastoma is the most common intraocular neoplasm of childhood. It comprises approximately 4% of all pediatric cancers, with greater than 95% of cases occurring before the age of 5. Approximately 40% of cases are hereditary, with *RB1* alterations. The majority of these patients present with bilateral disease with an average of three tumors per eye and present in the first year of life. The remaining 60% of cases are sporadic, most often presenting with unilateral and unifocal disease in the second and third years of life. Leukocoria is the most common presenting sign, followed by strabismus. Enucleation can be curative if the tumor has not spread to the choroid or optic nerve. For patients with heritable retinoblastoma, an eye-salvaging approach may be preferred, and traditionally have undergone chemoreduction with vincristine, carboplatin, and/or etoposide. Focal therapies such as plaque brachytherapy and external beam radiotherapy have long been used when the tumor is relatively small and confined to the retina [1].

While effective, these conventional therapies often lead to significant long-term morbidity and a decreased quality of life for pediatric patients. New drug delivery systems have circumvented some of these side effects [2]. However, targeting specific components of the retinoblastoma tumor cells and their microenvironment offer the chance to eradicate the neoplasm with the least amount of side effects. Immunotherapy holds much promise for patients with retinoblastoma, highlighting the need to understand the molecular and cellular specializations of the neoplasm and its associated immune microenvironment.

## 2. Breaking Down Retinoblastoma—The Neoplastic Cells

**Figure 1:**
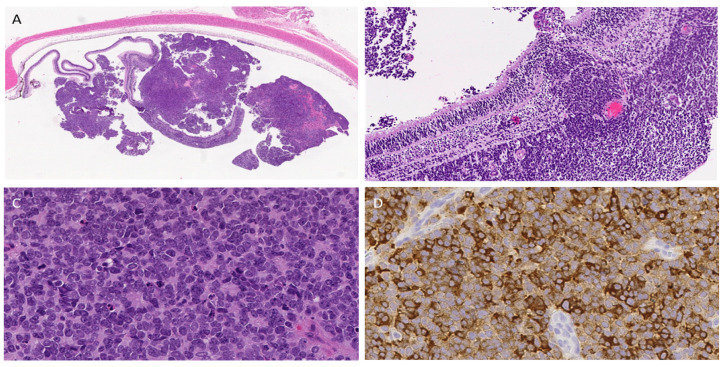
Histopathology of retinoblastoma. (**A**). A low-magnification photomicrograph of an enucleation stained with hematoxylin and eosin shows a malignant neoplasm emanating from the retina. Fragments of unattached tumors in this example are mainly due to tissue processing. There is no tumor in the sclera or the tissue outside the sclera in this example. (**B**) An intermediate-magnification photomicrograph shows regional tumor heterogeneity in the retina, with a large nodule of tumor extending through the outer retina. (**C**) A high-magnification photomicrograph shows a primitive neoplasm with numerous Homer-Wright rosettes. Scattered mitoses are present. (**D**) Many of the tumor cells express neuron-specific enolase (NSE). Figure 1 was created using Microsoft Publisher 2021 for layout and editing. Ethical review and approval were waived because the images came from our Dartmouth institutional archive and did not meet the US Food and Drug Administration or Department of Health and Human Services criteria for human research.

Retinoblastomas are heterogeneous masses arising from the retina (Figure 1A) that infiltrate and can invade the vitreous (Figure 1B). The hyperchromatic tumor cells have scant cytoplasm and are sometimes arranged in Homer-Wright rosettes, with lumina composed of cytoplasmic tumor cell processes (Figure 1C), or in Flexner–Wintersteiner rosettes or fleurettes. The cell of origin of retinoblastoma has been the subject of intense debate for at least 150 years, with growing consensus that the neoplasm arises from cone photoreceptor precursors with inactivation and loss of *RB1* [3,4]. Other important recurrent genomic abnormalities in tumor cells include *MYCN* and *MDM4* amplification [5]. Tumor cells have long been known to express neural markers such as neuron-specific enolase (Figure 1D).

Several tumor-associated antigens are known to be expressed in retinoblastoma (Table 1). B7-H3/CD276 was reported to be highly expressed in retinoblastomas in a heterogenous layout: high concentration in some tumor lobules with lower expression in adjacent blood vessels, and vice versa [6]. The relative abundance of B7-H3/CD276 in blood vessels and in the outer nuclear layer may limit the use of B7H3-targeted therapies in retinoblastoma. While B7-H3/CD276 was increased in poorly differentiated and non-neural invasive tumors, there was no difference in expression between low-risk and high-risk tumors [6]. Several groups have found that GD2, a disialoganglioside of glycolipid subtype, is highly expressed in retinoblastoma with limited expression in retina [7,8,9,10]. In addition, several cell adhesion molecules, such as L1-CAM/CD171 [7], glypican-2 [11], and EpCAM [12], are more highly expressed in retinoblastoma compared to retina. EpCAM, in particular, was found to be higher in invasive and poorly differentiated tumors compared to non-invasive and well-differentiated tumors. Finally, the SYK tyrosine kinase is aberrantly expressed in retinoblastoma [13]. Given the differential expression patterns of these antigens in retinoblastoma compared to retina, they may represent important targets for immunotherapies (see below).

Several proteins were identified in the retinoblastoma cell secretomes and aqueous humor that are also known to have immunosuppressive effects, including cytokines. Key proteins identified included those involved in inhibiting T cell activity and promoting immune tolerance, which contribute to the immune-suppressive environment in retinoblastoma. Aqueous humor analysis of retinoblastoma patients also shared elevated levels of immunosuppressive cytokines and soluble factors that can affect local immune responses in the eye. The concentrations of these factors correlated with disease stage and severity, indicating their potential role in disease progression [14].

With the administration of chemotherapy, several cell survival programs become activated in the neoplastic cells [15]. The long non-coding RNA *UCA1* promotes chemoresistance by sequestering miR-513a-5p, thereby increasing stathmin1 [16] and by increasing c-Met and AXL expression [17]. Activation of PI3K-AKT signaling pathways [15], such as through downregulation of the putative chemoresistance biomarker ARHGAP9 [18], may prevent apoptosis and/or promote DNA repair. Chemoresistant tumors show higher expression of cancer stem cell markers like SOX2, NANOG, OCT4 [19]. Finally, several studies have shown the increased presence in chemoresistant retinoblastomas of ATP-binding cassette transporters such as ABCB1 [15,16,19,20,21]. Pharmacologic inhibition of efflux transporters has been shown to decrease chemoresistance [15]. Targeting these and other biomarkers, potentially via novel drug delivery systems [22,23], represent possible counter-strategies to overcome chemoresistance in retinoblastoma.

## 3. Breaking Down Retinoblastoma—The Stromal Cells

Astrocytes [24] and fibroblasts [25] in the retinoblastoma microenvironment play a significant role in shaping retinoblastoma growth and heterogeneity [24,26]. These stromal cells contribute to the structure and behavior of the tumor, influencing how it develops and responds to treatment. Astrocytes, for example, have been found within retinoblastoma tumors and are believed to help the neoplastic cells survive by releasing factors that support tumor growth [24]. Their presence adds to the complexity of the tumor and supports the idea that retinoblastoma is not made up of tumor cells alone, but a mix of different cell types working together to support disease progression [26]. These stromal components may help explain the transcriptional and spatial heterogeneity seen in retinoblastoma [5]. In other words, the differences seen between and within tumors— like varying gene expression or cell types—might be partly due to the influence of these non-neoplastic cells. Understanding how astrocytes and fibroblasts contribute to retinoblastoma could open up new therapeutic targets aimed at disrupting these interactions.

Retinoblastoma is a highly vascular tumor. Neoplastic cells initially appear to utilize and grow along pre-existing retinal blood vessels. Eventually, the tumor contains new blood vessels that sometimes lack an intact blood–retinal barrier. Immune system cells, including macrophages, monocytes, dendritic cells and lymphocytes, are often present adjacent to blood vessels in retinoblastoma [27].

## 4. The Immune Microenvironment

**Figure 2:**
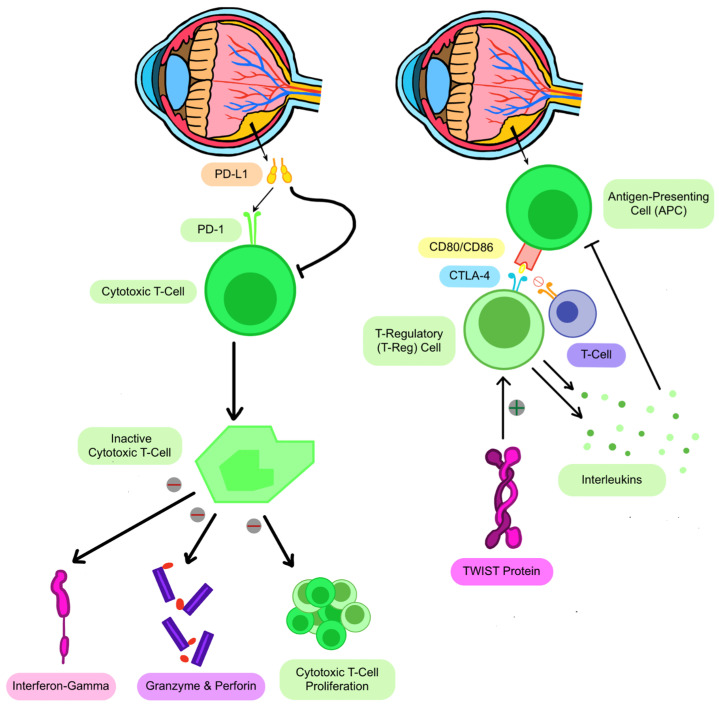
Immune evasion mechanisms in retinoblastoma and therapeutic targets. Biological cancer markers native to the human body contribute to retinoblastoma (RB) progression by suppressing immune responses. Programmed cell death protein 1 (PD-1), expressed on T cells, inhibits T cell activity upon interaction with its ligand PD-L1, which is often upregulated by tumors to evade immune detection. This interaction suppresses immune functions such as IFN-γ secretion, granzyme and perforin production, and T cell proliferation. Similarly, CTLA-4 receptors on T cells act as negative regulators of immune activation. Regulatory T cells (Tregs), which maintain immune homeostasis, are increased in RB due to increased Twist protein levels, leading to immune suppression. Figure 2 was created using Notability 15.3.3.

Analyzing the tumor immune microenvironment at the cellular and molecular level [28] provides insight into the various participants in retinoblastoma (Figure 2). Programmed cell death protein 1 (PD-1) is expressed on T cells, and negatively regulates T cell activity. The ligand of PD-1 (PD-L1) is expressed by tumors at times to exploit the immunosuppression pathway by interacting with, and inhibiting, PD-1 receptors on T cells. This, in turn, inhibits interferon-gamma (IFN-*y*) secretion, granzyme and perforin production, and T cell proliferation. To combat this, PD-1 antagonists, such as nivolumab, can be administered to block interactions between PD-1 and PD-L1 and inactivate immunosuppressive activity, allowing T cells to function normally. In a similar fashion, CTLA-4 on the surfaces of T cells acts as a negative regulator to prevent over-activation. Regulatory T cells (T-regs) maintain immune homeostasis and prevent autoimmune diseases by suppressing excessive or inappropriate immune responses. In RB patients, Twist protein concentrations are increased, leading to increased production of T-regs and drastic hypofunction of the immune system [29]. Although macrophages are typically known to destroy pathogens, Type II macrophages (M2) also contribute to immune suppression, helping tumors evade detection.

The immune system combats retinoblastoma through both innate and adaptive immune responses (Table 2). Macrophages, specifically type I macrophages (M1), play a key role in early tumor surveillance by engulfing antigens and releasing proinflammatory signals (mainly cytokines). Natural killer (NK) cells provide rapid cytotoxic activity by secreting granules containing perforin and granzymes, inducing apoptosis in neoplastic cells. Dendritic cells, as antigen-presenting cells (APCs), capture tumor antigens and present them to T cells bridging the innate and adaptive immune responses. Once dendritic cells present tumor antigens, T cells orchestrate a targeted immune attack. Cytotoxic T cells (CD8+) recognize tumor-specific antigens displayed on MHC class I molecules and secrete perforin and granzyme B, leading to cancer cell apoptosis. Helper T cells (Th) further regulate the response by rallying other immune cells upon encountering an antigen: Th1 cells activate cytotoxic T cells and macrophages for direct tumor killing, while Th2 cells stimulate B cells to differentiate into plasma cells that produce antibodies against tumor antigens.

Building on the critical role of the immune system in combating retinoblastoma, recent research highlights how chemotherapy can significantly alter the tumor’s immune microenvironment. In a typical treatment-naive setting, the tumor environment is immune-suppressive, with low CD8+ T cell infiltration [14,30]. PD-L1 expression in treatment-naïve tumors is also generally low [14,30] although one group identified PD-L1 expression in 31.94% of tumors [33]. Chemotherapy has been shown by some groups to shift this immune-cold environment, increasing CD8+ T cell presence [30] and upregulating PD-L1 expression [30,33], creating a more improved immune surveillance, allowing the immune system to better recognize and destroy cancer cells. Together, these findings suggest that combining chemotherapy with immunotherapies could provide a more effective strategy for treating retinoblastoma by altering the immune microenvironment. It should be noted, however, that one group did not find a change in cytotoxic T cells, tumor-associated macrophages (M2) or PD-L1 expression with chemotherapy [14].

## 5. Current Immunotherapy

Current immunotherapy strategies leverage monoclonal antibodies such as dinutuximab, pembrolizumab, ipilimumab, and nivolumab to enhance anti-tumor immune responses, particularly through combinatorial approaches that optimize both innate and adaptive immunity [34,35,36,37]. Dinutuximab, an anti-GD2 antibody, demonstrated potential in treating retinoblastoma by enhancing NK cell-mediated cytotoxicity of retinoblastoma cell lines via the perforin-granzyme B pathway [34]. Other cytotoxic mechanisms include complement activation and a mitochondria-dependent pathway [38]. Importantly, administration of dinutuximab beta (an analog of dinutuximab) in patients with metastatic retinoblastoma after autologous stem cell transplant either singly [39,40] or in combination with other immunotherapies [41] has led to clinical response and often prolonged survival.

Meanwhile, immune checkpoint inhibitors like pembrolizumab and nivolumab work by blocking PD-1, a receptor that suppresses T cell activity, thereby reinvigorating cytotoxic T lymphocytes to attack tumors. Similarly, ipilimumab, which is an anti-CTLA-4 antibody, promotes T cell activation by preventing inhibitory signals that would otherwise dampen the immune response [25]. Given that chemotherapy has been shown to increase PD-L1 expression in retinoblastoma, the combination of checkpoint inhibitors with traditional treatments may enhance immune-mediated tumor clearance [33]. Incorporating dinutuximab to boost NK cell cytotoxicity alongside checkpoint inhibitors to sustain T cell activation may provide a synergistic effect, overcoming immune evasion mechanisms. These combinatorial strategies highlight the future potential of integrating antibody-based immunotherapies with conventional treatments to improve retinoblastoma outcomes. A significant hurdle to overcome is the variable presence of the blood–retina barrier and other anatomic factors that may diminish these drugs from reaching the retinoblastoma tumor cells.

## 6. Future Directions and Implications for Cell-Based Therapies

Spatial omics technologies [42] have shed light on how tumors are organized and what types of cells they contain. This is beneficial for understanding of retinoblastoma, where it is difficult to separate tumor cells from normal retinal or immune cells in bulk samples. Spatial whole transcriptome analysis of a retinoblastoma excision specimen using digital spatial profiling (DSP) revealed neighborhoods of poorly differentiated neoplasm adjacent to neighborhoods of well differentiated neoplasm [43]. Newer technologies allow for cellular and subcellular resolution [44], which could help identify new targets for treatment.

One promising area of therapy involves utilizing CAR-T cells, which are genetically modified immune cells designed to attack specific proteins found on cancer cells. In retinoblastoma, CAR-T cells have been generated against CD171, GD2, and glypican-2, and these cells have shown efficacy in preclinical studies [7,8,9,11]. DSP and single-cell RNA sequencing have also revealed key genes like *UBE2C* and *SOX4* that seem to drive tumor proliferation and invasion, making them possible targets for therapies like CAR-T [5]. These tools and approaches offer a more detailed understanding of the tumor and support the development of targeted, immune-based treatments that could improve outcomes for retinoblastoma patients.

## Figures and Tables

**Table 1:** Expression of tumor-associated antigens in human retinoblastoma.

Protein	Tissue Analyzed	Technique	Findings	Reference
B7-H3/ CD276	Primary retinoblastomas, postmortem adult retinas.	Western blot, immunohistochemistry.	Increased in poorly differentiated, non-neural invasive tumors. No difference between low-risk and high-risk tumors. Expression in normal outer nuclear layer.	[6]
L1CAM/ CD171	Retinoblastoma cell lines, primary retinoblastomas.	Flow cytometry, immunohistochemistry.	Expressed in all retinoblastoma cell lines and in half of primary retinoblastomas.	[7]
GD2	Retinoblastoma cell lines.	Flow cytometry.	Expressed in all retinoblastoma cell lines.	[7]
GD2	Retinoblastoma cell line, retina cell line, primary retinoblastomas.	Flow cytometry, immunohistochemistry.	Expressed in all primary retinoblastomas and in the retinoblastoma cell line, but not in the retina cell line.	[8]
GD2	Retinoblastoma cell lines, primary retinoblastoma.	Flow cytometry, immunohistochemistry.	Expressed in primary retinoblastoma and the two retinoblastoma cell lines, but not in normal human retina.	[9]
GD2	Primary retinoblastomas, serum samples from retinoblastoma patients and healthy individuals.	Thin layer chromatography and immunoblot.	Second most abundant disialoganglioside in primary retinoblastomas. Significantly elevated in serum of patients with retinoblastoma.	[10]
Glypican-2	Primary retinoblastomas, retinoblastoma cell lines, retina cell line	Flow cytometry, immunohistochemistry.	Expressed in all primary retinoblastomas, including metastases, but not in adjacent uninvolved retina. More highly expressed in retinoblastoma cell lines compared to retina cell line.	[11]
EpCAM	Primary retinoblastomas.	Immunohistochemistry.	Higher in invasive and poorly differentiated tumors compared to non-invasive and well-differentiated tumors. Expressed in outer nuclear layer, inner nuclear layer and ganglion cell layer of non-neoplastic retina.	[12]
SYK	Retinoblastoma cell line, retina cell line.	Western blot, immunofluorescence, qRT-PCR.	Upregulated in retinoblastoma cell line compared to retina cell line.	[13]

**Table 2:** Components of the immune microenvironment in untreated human retinoblastoma.

Cell Types Present	Cell Types Very Low or Absent	mRNAs and/or Proteins Present	mRNAs and/or Proteins Very Low or Absent	Reference
Tumor-associated macrophage (M2), cytotoxic T cell, regulatory T cell.	Not reported	EMMPRIN, MIF	PD-L1 (95% of tumor samples)	[14]
Tumor infiltrating immune cells were observed, mostly within large GFAP+, PD-L1+ gliotic areas, in 11.4% of cases.	All tumor infiltrating immune cells were sparse and found in perivascular and perinecrotic areas.	Perivascular and intratumoral glial cells expressed PD-L1. PD-L1 and PD-1 positivity increased in retinal areas closest to neoplasm. In 20.5% of cases, patchy PD-1 was observed in neoplastic cells.	Few neoplastic cells near necrotic areas showed PD-L1 positivity. PD-L1+ and PD-1+ tumor infiltrating immune cells were very rare.	[30]
28 different types, with consistent presence of T cells and myeloid lineage cells. Some tumors (lower tumor size) had high immune infiltration.	Low immune infiltration in tumors with increased proliferation and metastasis.	Not reported.	Not reported.	[31]
Increased CD45+ immune infiltration (more than 5%) in tumors with lowest Ki67 levels.	Decreased CD45+ immune infiltration (less than 5%) in tumors with highest Ki67 levels.	Not reported.	Immune infiltrate gene signature present at lowest levels in highly proliferative retinoblastoma cell lines.	[32]
Not reported.	Not reported.	PD-1 in 20.13%, PD-L1 in 30.94%. CTLA-4 in 31.9% of tumor cells.	Not reported.	[33]
T cells, B cells.	NK cells.	Complement, inflammatory response and IL6/JAK/STAT3 gene set pathways.	Pathways involved in biosynthesis and secretion of cytokines, innate immune, NK cell immune modulation, stimulatory natural cytotoxicity receptors, and perforin-granzyme were deregulated.	[34]
Tumor-associated macrophages, with shift from M1 to M2 type during invasion.	Very low numbers of T cells, NK cells and B cells.	Tumor-associated macrophage signaling pathways (like GRN and MIF), and chemokines like CCLs and galectins.	Lymphocyte-associated proteins.	[28]
Regulatory T cells (8% of total tumor cells by flow cytometry).	Not reported.	Not reported.	Not reported.	[29]

## Data Availability

No new data were created or analyzed in this study.
